# Food allergy in preschool children in Qingdao, China: a cross-sectional study

**DOI:** 10.3389/fped.2026.1852387

**Published:** 2026-06-26

**Authors:** Chuanyue Qiao, Lin Qi, Jie Guo, Rui Han, Lei Ma

**Affiliations:** 1 Department of Stomatology, Traditional Chinese Medical Hospital of Huangdao District, Qingdao, China; 2 Department of Stomatology, The First Affiliated Hospital of Dali University, Yunnan, China; 3 Department of Prosthodontics, The Affiliated Hospital of Qingdao University, Qingdao, China

**Keywords:** allergy, food allergy, influencing factors, pediatrics, preschool children

## Abstract

**Background:**

Food allergy (FA) is a common childhood condition, and its global prevalence has risen over the past two decades.

**Methods:**

A cross-sectional study was conducted between April and July 2025, involving 461 preschool children in Qingdao, China. Data were collected by means of a self-administered questionnaire completed by parents or guardians.

**Results:**

Among the 461 participating preschool children, 60 (13.0%) were diagnosed with FA. No statistically significant differences in FA prevalence were observed across gender or age groups. Of the FA-affected children, 35 (58.3%) exhibited a single clinical manifestation, while 25 (41.7%) presented with two or more concurrent symptoms. Univariate analysis identified significant associations between FA occurrence and the following factors: annual household income, gestational age at birth (weeks), maternal FA history, child's picky eating behavior, and indoor plant cultivation. Binary logistic regression analysis further revealed that: 1. Risk factors for FA: Annual household income > ¥15,000 CNY [OR (95% CI): 2.812 (1.547-5.111)], maternal FA history [OR (95% CI): 3.143 (1.505-6.561)], and picky eating behavior [OR (95% CI): 2.558 (1.383-4.732)]. 2. Protective factors against FA: Gestational age ≥ 37 weeks [OR (95% CI): 0.049 (0.006-0.424)] and indoor plant cultivation [OR (95% CI): 0.433 (0.229-0.817)].

**Conclusions:**

A high prevalence of FA was identified among preschool children in Qingdao, China. The most common allergenic foods included shrimp, mango, milk, and eggs. Cutaneous symptoms were the predominant clinical manifestations associated with FA. The risk factors for FA were multifactorial. An annual household income exceeding ¥150,000, a maternal history of FA, and picky eating behavior were identified as significant risk factors. In contrast, a gestational age of 37 weeks or more and indoor plant keeping were associated with a reduced risk of FA.

## 1 Introduction

Food allergy (FA) is a reproducible adverse immune response to specific food antigens, which may be immunoglobulin E (IgE)-mediated or non-IgE-mediated (1). This immune response is rapid, with symptom onset typically occurring within 5–60 min following exposure to the food. FA is a severe, life-threatening allergic reaction characterized by rapid onset and potential fatality. It involves multiple organ systems, including the respiratory tract, gastrointestinal tract, and skin (2).

The most critical step in diagnosing FA is obtaining a comprehensive clinical history, including the type of food ingested, the nature of the symptoms, and the timing of the reaction (3). Subsequent confirmation may be achieved through SPT (skin prick testing) or measurement of allergen-specific IgE antibodies (4). However, these methods have a high false positive rate (5). Although the double-blind, placebo-controlled food challenge remains the gold standard for diagnosis, its associated risk of anaphylaxis makes it unsuitable for routine use in large-scale or intensive testing protocols.

According to epidemiological studies, FA affects approximately 8% of children and 3% of adults in Europe, the United States, and other industrialized regions (6). The most prevalent allergens include cow's milk, eggs, peanuts, soybeans, nuts, fish, and crustaceans—along with their derived products. These eight categories, designated by the World Health Organization as the “Big Eight”, account for over 90% of all documented food allergic reactions (7). The global incidence of allergic diseases is increasing, underscoring the urgency of addressing FA, especially among children (8). FA symptoms in children are often severe and can persist into adulthood. Studies report that the prevalence of FA in children rose from approximately 1.3% in 1997 to between 5.5% and 7.3% in 2012, and has exceeded 8% in the past decade (9). According to uncompleted statistics, the prevalence of FA is approximately 8.8% in Chengdu (10), 7.58% in Panzhihua (11) and 11.7% in Taiyuan (12). Clinical manifestations of FA are frequently nonspecific, which may result in misdiagnosis or underdiagnosis, thereby jeopardizing children's health (13). In severe instances, FA can impair growth and development and may even lead to fatal anaphylactic shock (13).

FA likely arises from impaired or delayed development of oral tolerance—or from the absence of clinical reactivity to a food antigen—in individuals who are genetically, and potentially environmentally, predisposed to atopic disease (14). Heredity plays a significant role in the development of FA. For example, Rajani et al. and Di et al. suggest that maternal mode of delivery, feeding patterns, and mood during pregnancy influence the risk of food allergy, potentially by affecting the composition of the gut microbiome in early life (15, 16). In addition, various lifestyle-related risk factors are associated with the development of FA. For instance, increased environmental exposure to animal fur, plant pollen, and air pollutants may contribute substantially to the rising incidence of FA (17).

In summary, the prevalence of FA is increasing, imposing a substantial burden on public health and quality of life. Nevertheless, nationwide epidemiological data on FA remain scarce, particularly for the city of Qingdao. Therefore, a cross-sectional survey is warranted to elucidate the current status of FA. Such an investigation is crucial for the early diagnosis of FA in children, the identification of its risk factors, the improvement of clinical prevention, management, and treatment strategies, and ultimately, the promotion of healthy child development.

## 2 Materials and methods

### 2.1 Study participants

Qingdao is a major central city on the eastern coast of China, with a permanent residential population of 10.555 million, of whom approximately 15.41% (about 1.552 million) are children aged 0–14 years. Given the large pediatric population, children's health is a public health priority. Furthermore, Qingdao has a highly developed economy, a large and diverse food consumption market, and an abundance of seafood products in particular. Consequently, local children have greater opportunities for exposure to food allergens. Thus, conducting this survey among preschool children in Qingdao offers both strong regional representativeness and substantial practical research value.

### 2.2 Study design and setting

From April to June 2025, we selected one public kindergarten in each of the six main administrative districts of Qingdao (Shinan, Shibei, Licang, Laoshan, Huangdao and Chengyang District) by simple random sampling method. All children aged 3–6 years old within these kindergartens were then included as study participants. This population group is in a critical stage of physical growth and dietary habit formation, and is also a high-risk group susceptible to food allergy, which is suitable for exploring the epidemiological characteristics and influencing factors of childhood food allergy. The inclusion criteria were as follows: (1) aged 3–6 years; (2) absence of systemic diseases. The exclusion criteria included: (1) presence of systemic diseases; (2) inability of parents or guardians to cooperate in completing the survey. A total of 461 children were finally screened.

### 2.3 Ethics approval

This investigation was approved by the Ethics Committee of The Affiliated Hospital of Qingdao University (QDFYQN202101020), and informed consent was obtained from the legal guardians of enrolled children.

### 2.4 Questionnaire

The study objectives were thoroughly explained by the investigators to the principals and teachers of the participating kindergartens. Furthermore, it was assured that all questionnaire data would remain strictly confidential and inaccessible to individuals outside the research team. Upon obtaining approval from both the schools and the children's parents or legal guardians, an on-site questionnaire survey was administered. The questionnaires were distributed in a private setting, and no third parties other than research team members and respondents were present during the collection process. All questions, including those pertaining to FA, were completed by parents or guardians under the guidance of trained investigators.

Each questionnaire was accompanied by a copy of the informed consent form. A self-designed questionnaire, adapted from the EuroPrevall FA survey (18) with context-specific modifications, was used. The questionnaire included single-choice questions, multiple choice questions, and subjective questions. It consisted of 27 items covering key domains such as: basic demographic information of the child and family, living and dietary habits, home environment, early feeding practices, birth history, environmental exposures, genetic background, and food allergy history (Supplementary Table S1). FA diagnoses included both self-reported and medically confirmed cases. For self-reported FA, the question was phrased as: “Has your child ever experienced symptoms of a food allergy?” with binary response options (yes/no). For medically diagnosed FA, the question was phrased as: “Has your child ever been medically diagnosed with a food allergy?” with binary response options (yes/no). Participants who answered “yes” were asked to provide a complete medical diagnosis certificate.

All investigators involved in questionnaire administration and data entry received standardized training. Furthermore, we randomly selected 30 valid participants for retesting, who completed the same questionnaire again after a two-week interval. The consistency rate between the two rounds of survey responses reached 98.5%.

### 2.5 Statistical analysis

Statistical analysis was performed using Excel (Microsoft, Redmond, Washington, USA) and SPSS 23.0 (IBM, Armonk, New York, USA). Categorical variables were expressed as *n* (%) and statistically analyzed using the Chi-square (*χ*^2^) test or Fisher's exact Test. The factors with statistical significance in the univariable analyses were included as independent factors in multivariable binary logistic regression model. Associations between risk factors and FA were expressed as odds ratio (OR) with 95% confidence intervals (CI). A *p*-value < 0.05 was considered statistically significant.

## 3 Results

### 3.1 Participants and prevalence of FA

A total of 461 questionnaires were distributed to parents or guardians, and all 461 were returned, resulting in a response rate of 100%. The study population consisted of 248 boys (53.8%) and 213 girls (46.2%), with a mean age of 4.8 ± 1.1 years. The prevalence of self-reported FA was 18.9% (87/461), and the prevalence of medically diagnosed FA was 13% (60/461). Any of the following FA refer to medically diagnosed FA. No significant difference in FA prevalence was observed between males and females or across different age groups (*p* > 0.05; see Table 1).

**Table 1:** Prevalence of FA according to gender and age.

Variables	Total number	FA (%)	No-FA (%)	*χ^2^*	*P*
Gender					
Female	213	25 (11.7)	188 (88.2)	0.57	NS
Male	248	35 (14.1)	213 (85.8)		
Age (years)					
3–4	58	8 (13.8)	50 (86.2)	2.85	NS
4–5	128	21 (16.4)	107 (83.6)		
5–6	103	14 (13.6)	89 (86.4)		
6–7	172	17 (9.9)	155 (90.1)		

FA, food allergy.

### 3.2 Symptoms in children with FA

**Figure 1:**
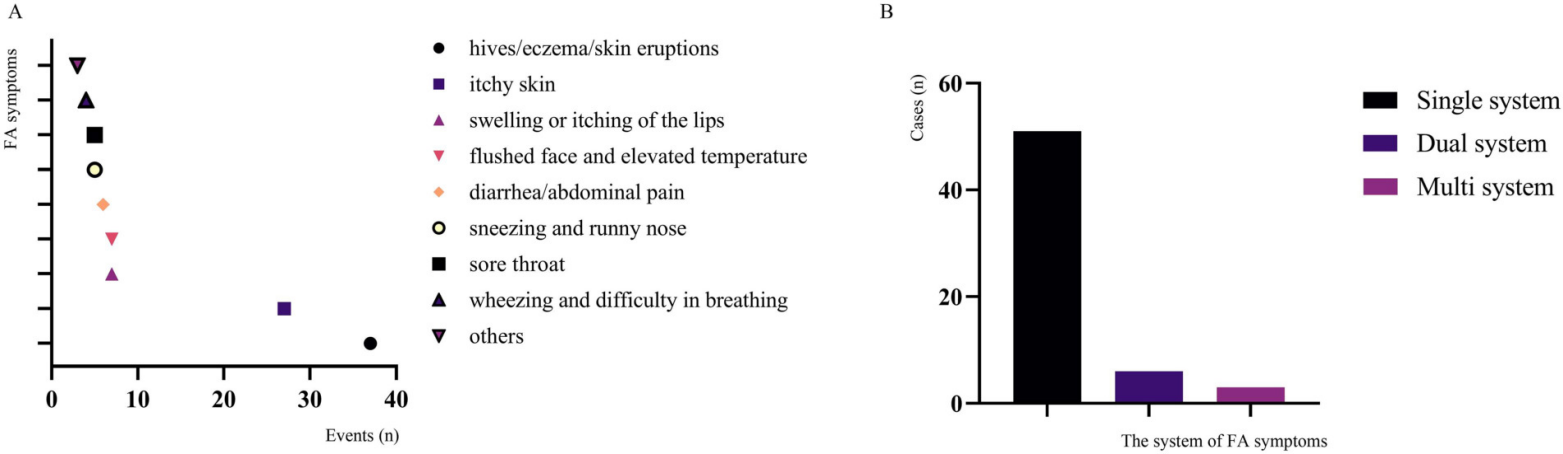
Occurrence of different FA symptoms. **(A)** The number of cases of different FA symptom events (n). **(B)** FA with single system symptoms, FA with two systems, FA with three or more systems (n).

The results of the study showed that skin abnormalities—such as hives, eczema, and skin eruptions—were the most common symptoms, with an event rate of 61.7% (37/60). The second most frequent symptom was itchy skin, affecting 45% (27/60) of the children. Swelling or itching of the lips and diarrhea/abdominal pain each occurred in 11.7% (7/60) of cases. Nausea, vomiting, or food refusal, along with flushed face and elevated temperature, were each observed in 8.3% (5/60) of the children. Additionally, sore throat was reported in 6.7% (4/60), sneezing and runny nose in 10% (6/60), and wheezing or difficulty breathing in 5.0% (3/60) of cases (Figure 1A).

Among the 60 children with FA, 51 exhibited clinical reactions involving only one organ system, with cutaneous manifestations being the most common and generally causing mild impact; 6 patients presented with clinical manifestations involving two organ systems; Three cases involved the skin, respiratory tract, and digestive tract, frequently accompanied by severe respiratory symptoms (Figure 1B).

### 3.3 The type of food intolerance

**Figure 2:**
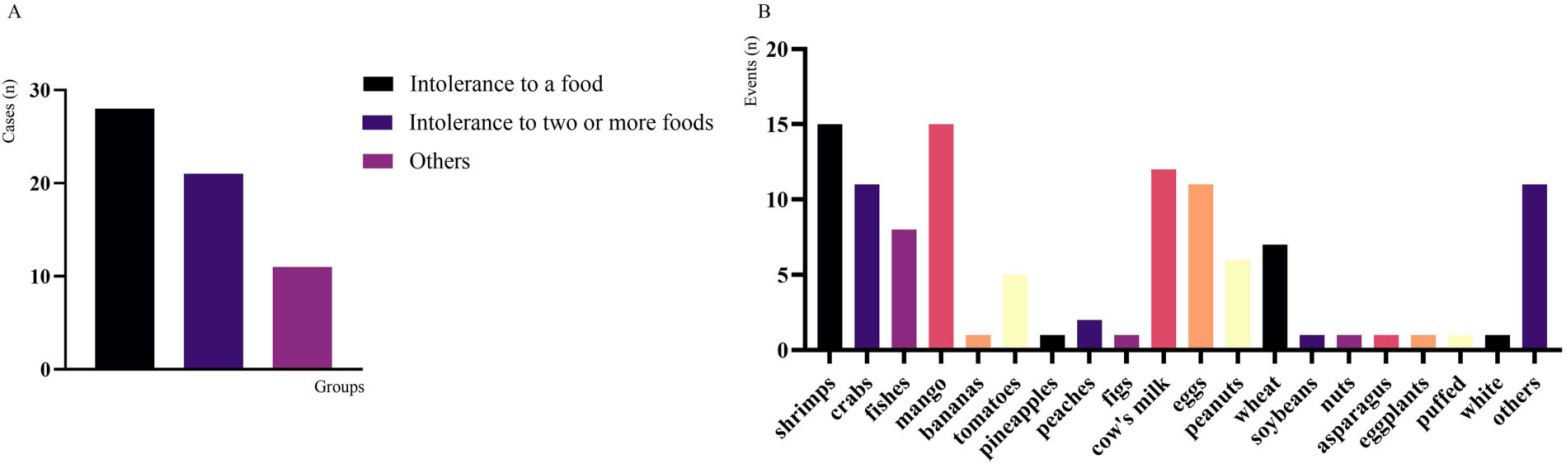
Types of food intolerances. **(A)** Number of children with one food intolerance and two or more food intolerance (n). **(B)** Number of different food allergens (n).

Among the 60 children diagnosed with FA, 28 were allergic to a single food item, while 21 exhibited allergies to two or more items (Figure 2A). The most common allergenic foods were seafood (including shrimp, crab, and fish) and fruits (such as mango, banana, tomato, pineapple, peach, and fig), affecting 34 and 25 children, respectively. These were followed by milk (12 cases) and eggs (11 cases). Allergies to wheat and peanuts were reported in 7 and 6 children, respectively. Additionally, one case each was reported for soybean, nuts, mushrooms, eggplant, puffed foods, and silver fungus allergies (Figure 2B).

### 3.4 Potential risk factors associated with FA

Univariate analysis identified several factors significantly associated with FA (Table 2). These included annual household income, gestational age, maternal history of FA, picky eating behavior, and indoor plant keeping (all *p* < 0.05).

**Table 2:** Potential risk factors associated with FA.

Variables	Total number	FA (%)	No-FA (%)	*χ^2^*	*p*
Annual family income (RMB)					
< 150000	260	23 (8.8)	237 (91.2)	9.155	0.002*
≥ 150000	201	37 (18.4)	164 (81.6)		
Childbearing age (years)					
< 25	36	2 (5.6)	34 (94.4)	/	NS
25 ≤ age < 30	200	28 (14.0)	172 (86.0)		
≥ 30	225	30 (13.3)	195 (86.7)		
Gestational age (weeks)					
< 37	40	11 (27.5)	29 (72.5)	/	0.005*
37 ≤ week < 42	385	48 (12.5)	337 (87.5)		
≥ 42	36	1 (2.8)	35 (97.2)		
Antibiotics at birth					
Yes	13	3 (23.1)	10 (76.9)	/	NS
No	448	57 (12.7)	391 (89.0)		
Mother with FA					
Yes	50	14 (28.0)	36 (72.0)	11.123	0.001*
No	411	46 (11.2)	365 (88.8)		
Mode of birth					
Eutocia	250	36 (14.4)	214 (85.6)	0.925	NS
Caesarean section	211	24 (11.4)	187 (88.6)		
Feeding pattern					
Breastfeeding	333	43 (12.9)	290 (87.1)	/	NS
Milk powder feeding	9	2 (22.2)	7 (77.8)		
Mixed feeding	119	15 (12.6)	104 (87.4)		
Only child					
Yes	102	16 (15.7)	86 (84.3)	0.825	NS
No	359	44 (12.3)	315 (87.7)		
Particular about food					
Yes	120	23 (19.2)	97 (80.8)	5.422	0.020*
No	341	37 (10.9)	304 (89.1)		
Residential floor					
1–3	157	15 (9.5)	142 (90.5)	3.246	NS
4–6	152	25 (16.4)	127 (83.6)		
7–9	46	6 (13.0)	40 (87.0)		
≥ 10	106	14 (13.2)	92 (86.8)		
Residence has intersection or factory					
Yes	229	30 (13.1)	199 (86.9)	0.003	NS
No	232	30 (12.9)	202 (87.1)		
Move into a new home or renovate it within 6 months					
Yes	34	2 (5.9)	32 (94.1)	/	NS
No	427	58 (13.6)	369 (86.4)		
Indoor plant					
Yes	357	40 (11.2)	317 (88.8)	4.583	0.032*
No	104	20 (19.2)	84 (80.8)		
Pet feeding					
Yes	43	6 (14.0)	37 (86.0)	0.037	NS
No	418	54 (12.9)	364 (87.1)		
Any smokers at home					
Yes	174	22 (12.6)	152 (87.4)	0.034	NS
No	287	38 (13.3)	249 (86.8)		

*p < 0.05, FA, food allergy.

### 3.5 Logistic regression analysis of risk factors for FA

The diagnosis of FA in infants and young children was defined as the dependent variable and coded as 1 (“yes”) or 0 (“no”). Five factors that showed significant associations in the univariate analysis—annual household income, gestational age, maternal history of FA, picky eating behavior, and indoor plant keeping—were included as independent variables in a binary logistic regression model. Multivariate analysis revealed that an annual household income ≥ ¥150,000, maternal history of FA, and picky eating behavior were identified as risk factors for FA. In contrast, gestational age ≥ 37 weeks and indoor plant keeping were protective factors (Table 3).

**Table 3:** The ORs and 95% CI of risk factors associated with FA.

Variables	*β* value	SE value	Wald *χ^2^*	OR	95% CI		*p*
					Lower	Upper	
Annual family income (RMB)							
< 150000	Reference			1			
≥ 150000	1.034	0.305	11.497	2.812	1.547	5.111	0.001*
Gestational age (weeks)							
< 37	Reference			1			
37 ≤ week < 42	−3.017	1.101	7.508	0.049	0.006	0.424	0.006*
≥ 42	−1.040	0.412	6.363	0.353	0.157	0.793	0.012*
Mother with FA	1.145	0.376	9.297	3.143	1.505	6.561	0.002*
Particular about food	0.939	0.314	8.960	2.558	1.383	4.732	0.003*
Indoor plant	−0.838	0.324	6.675	0.433	0.229	0.817	0.010*

*p < 0.05, FA, food allergy; OR, odds ratio; CI confidence interval.

## 4 Discussion

FA represents a common chronic condition affecting infants and young children. In recent decades, it has garnered increasing attention from families, clinicians, and policymakers worldwide. A meta-analysis indicated that 79.8% of FA cases emerge within the first year of life (19). In the United States, a 2018 population-based cross-sectional survey encompassing over 50,000 households estimated the prevalence of self-report FA to be approximately 10% in adults and 8% in children (20, 21). A recent Australian study reported a prevalence of 5.5% for self-reported FA and 4.5% for OFC (oral food challenge) diagnosed FA in children aged 10–14 years, alongside a rising incidence of hospital admissions due to food-induced anaphylaxis (22). Furthermore, a study conducted in Turkey, spanning a 20-year interval, revealed a substantial increase in the prevalence of allergic diseases (including FA) among children in 2022 compared to two decades prior (23).

FA represents a significant and growing public health concern globally, though epidemiological studies within China remain limited, particularly regarding pediatric populations in Qingdao. Among 461 children aged 3–6 years in Qingdao, the prevalence of self-reported FA was 18.9%, and the prevalence of medically diagnosed FA was 13.0% [95% CI: 10.1%–16.4%]. This figure is substantially higher than rates reported in many developed regions, including the United States and Australia, and also exceeds those documented in other Chinese cities. For example, a 2019 report from Chongqing reported a self-reported FA prevalence of 22% among 596 children and a medically diagnosed prevalence of 9.9% (24). A report from Guangdong Province showed that, among 5,542 children, the prevalence of medically diagnosed FA was 5.38% (25). Meanwhile, a study in Qinhuangdao reported that the prevalence of FA diagnosed by SPT (skin prick testing) was approximately 6.27% (26).

Worldwide, FA poses increasing several challenges for public health. The high prevalence of FA places a substantial burden on the healthcare system, including outpatient and emergency department visits, emergency treatment, and long-term follow-up. Moreover, severe allergic reactions may even endanger the life and health of children. On the other hand, FA also increases the burden on families, schools, the food service industry, and other social institutions, thereby imposing additional economic costs on both families and communities. These findings underscore the need for enhanced surveillance and regionally tailored public health strategies. Improving understanding of the etiology, distribution, and risk factors of FA in Qingdao will be essential for developing effective prevention and management protocols to mitigate its health impact.

The primary clinical manifestations of early FA commonly involve gastrointestinal and cutaneous symptoms, though multi-system involvement—including respiratory, immune, circadian, and neurological systems—may also occur (27). In the present study, cutaneous symptoms were particularly prominent, with urticaria, eczema, and related skin manifestations occurring in 61.7% of cases, and pruritus affecting 45.0% of affected children. Other frequently reported symptoms included perioral reactions, gastrointestinal disturbances, and nasal symptoms, with occasional episodes of dyspnea also documented.

Consistent with these findings, Feng et al. (2022) (28) reported that in Jiangxi Province, China, skin manifestations were the most common clinical feature (85.0%), followed by perioral symptoms (28.6%) and neurologic complaints such as headache and dizziness (18%) (28). In contrast, a study by Sasaki et al. based on oral food challenges in Australian children aged 10–14 years found that itching of the mouth or tongue was the most frequent symptom (47.0%), followed by cutaneous reactions (44.0%) (22). Despite variations in the reported prevalence of specific symptoms across studies, cutaneous and gastrointestinal manifestations consistently emerge among the most prevalent features of FA (29, 30).

Studies have shown that in developed Western countries—such as the United States, the United Kingdom, and Australia—the most prevalent food allergens among children aged one to five years are milk, eggs, and peanuts. Among those six years and older, peanuts represent the most common allergen, followed by shrimp, milk, and eggs (31). In contrast, our study identified a distinct allergen profile among children in Qingdao: seafood (including shrimp, crab, and fish) was the most frequently reported allergen, followed by mango, milk, eggs, wheat, and peanuts.

The etiology of FA involves a complex interplay of polygenic inheritance and environmental factors, which has attracted considerable research interest. Accumulating evidence underscores the significant role of genetic predisposition in early-onset FA (32). In alignment with this, our study specifically evaluated the influence of maternal allergic history on childhood FA. Both univariate and multivariate logistic regression analyses indicated that maternal atopy significantly increases the risk of FA in children. This finding is consistent with the report by Gelincik et al., which also documented a statistically elevated risk of FA among children with a family history of allergy (33). One proposed mechanism involves the placental transfer of inflammatory cytokines from allergic mothers, which may prime the fetal immune system and contribute to the development of FA in offspring (34). Prescott and Clifton also found that the maternal allergic phenotype are suggested to critically determine the risk of subsequent infant allergic disease through alterations in DNA and histone methylation, histone acetylation and chromatin structure (35). However, our study was an observational cross-sectional study. The specific biological mechanism is not clear.

Environmental factors exert considerable influence on the development of FA. Although early pet exposure has been proposed as a protective factor against allergic diseases—as reported by Okabe et al.—our study did not corroborate this effect. This discrepancy may arise from variability in pet species and domestic exposure contexts, which appear to modulate the immunomodulatory potential of pet ownership (36). In addition, tobacco smoke and airborne particulate matter have been implicated in the sensitization to common allergens, potentially elevating individual susceptibility to allergic conditions, as underscored by Feleszko et al. (37).

Our multivariate logistic regression identified several determinants significantly associated with FA, including higher annual household income (≥¥150,000), gestational age, picky eating behavior, and the presence of indoor plants. Notably, children from households with higher socioeconomic status exhibited an increased risk of FA—a finding consistent with observations by Gorris et al. (38). Parallel evidence comes from Davoodi et al., who documented a strong socioeconomic gradient in allergic asthma, with prevalence rates highest among upper-class (88.2%) compared to lower-income cohorts (34%) (39). Proposed mechanisms for this association include a higher propensity for cesarean delivery among affluent families, which may alter immune programming through modified inflammatory signaling and shifts in allergy-relevant cell populations, ultimately predisposing children to atopic disorders (38, 40). On the one hand, economic level may influence the mode of delivery chosen by mothers (41). Improvements in the healthcare system and increased disposable income have led to a rising number of mothers opting for cesarean section, which in turn affects the subsequent development of allergic diseases (38). On the other hand, some studies suggest that economic level may affect the occurrence of allergic diseases through factors such as exposure to indoor allergens, smoking, and air pollution levels. Therefore, economic level likely serves as a surrogate marker (42).

Perinatal factors significantly influence the development of allergic diseases in children, with preterm birth representing a well-established risk factor. In a meta-analysis incorporating 17 studies, Been et al. demonstrated that preterm infants face a 1.46-fold increased risk of asthma or childhood wheezing, while very preterm children (gestational age < 32 weeks) exhibit a threefold higher asthma risk compared to term-born counterparts (43). Consistent with these findings, our study identified gestational age ≥ 37 weeks as a protective factor against FA. The etiology of preterm birth remains multifactorial and incompletely elucidated, influenced by meteorological conditions, socioeconomic and racial disparities, genetic susceptibility, environmental exposures (e.g., air pollution and tobacco smoke), and individual behaviors such as maternal smoking and hygiene practices (44–46). Preterm delivery is associated with immaturity of the respiratory, digestive, and immune systems, which may enhance vulnerability to adverse environmental stimuli and predispose children to allergic conditions, including FA (47, 48). This developmental immaturity may underlie the higher prevalence of allergic diseases observed in preterm populations.

Environmental factors play a critical role in the occurrence of FA. Previous studies have indicated that exposure to air pollutants, such as sulfur dioxide and PM2.5, is associated with an increased prevalence of allergic diseases among children (40, 49). In the present study, the presence of indoor plants was identified as a protective factor against FA. Plants mitigate indoor air pollution by oxidizing highly toxic sulfur dioxide into less toxic sulfate compounds through leaf absorption and by reducing carbon dioxide levels while releasing oxygen via photosynthesis, thereby improving overall air quality (50, 51). These mechanisms suggest that improved indoor air quality, facilitated by household vegetation, may contribute to a reduced risk of FA.

This study presents preliminary data on the prevalence and associated factors of FA among preschool children in Qingdao, although several limitations should be acknowledged. First, the diagnosis of FA in this study was based on medical diagnosis certificates. However, there is heterogeneity in diagnostic standards and practices across medical institutions. A rigorous diagnosis of FA typically requires comprehensive consideration of clinical history, SPT, serum-specific IgE measurement, and OFC testing. This diagnostic heterogeneity may introduce classification bias, thereby affecting the accuracy of prevalence estimates. Addressing this issue will be a key focus of our subsequent research. Standardizing diagnostic criteria depends on collaborative efforts between pediatric healthcare and rheumatology departments to conduct more systematic investigations—a goal we are currently pursuing. Second, the sample size was relatively limited, which may reduce statistical power and compromise the stability of the results. Although we enrolled 461 children, subgroup analyses involved small cell counts, leading to wide confidence intervals and an increased risk of chance findings. Third, the study has regional limitations. Environmental exposures, dietary patterns, and the distribution of healthcare resources vary across regions. Consequently, the prevalence estimates and identified risk factors from this study may not be directly generalizable to other cities in China or to other countries. Fourth, the cross-sectional design precludes causal inference and lacks in-depth exploration of underlying mechanisms. Although we cite mechanistic hypotheses from the literature in the Discussion section, the present study alone cannot verify the mechanisms of FA. Future prospective cohort and experimental studies are needed to confirm causality and elucidate pathways.

However, as a preliminary study, we have obtained several instructive findings. First, Mother with FA and Gestational age were identified as factors associated with an increased risk of FA. In early clinical practice, children with these characteristics can be considered key monitoring targets. Clinicians should actively inquire about food-related adverse reactions during routine physical examinations and provide early guidance on dietary introduction. Second, educating parents to recognize allergic symptoms and their severity—such as sudden onset of generalized urticaria, swelling of the lips or eyelids, dyspnea, and persistent vomiting—is essential for timely pediatric healthcare seeking. Finally, although evidence for factors such as houseplants remains limited, general health recommendations may still have a positive effect on reducing allergy risk. These include maintaining a clean home environment, avoiding indoor tobacco exposure, encouraging reasonable outdoor activity, and using antibiotics prudently.

In summary, based on the current level of evidence, clinical practice should emphasize accurate diagnosis, avoidance of overtreatment, monitoring of high-risk children, and reduction of unnecessary fear and nutritional risk through standardized parental education. Future prospective intervention studies are needed to validate the actual effects of the above recommendations.

## 5 Conclusions

This study revealed a high prevalence of FA among preschool children in Qingdao, China. The predominant allergenic foods included seafood, mango, milk, and eggs, with cutaneous manifestations representing the most frequently reported symptoms. The etiology of FA involves multiple interacting factors, underscoring the complex nature of its development. A comprehensive understanding of the epidemiological characteristics and risk factors of FA is essential for guiding effective public health interventions and improving strategies for the prevention and clinical management of this condition.

## Data Availability

The original contributions presented in the study are included in the article/Supplementary Material, further inquiries can be directed to the corresponding author.
