# Supplementary material for: Food allergy in preschool children in Qingdao, China: a cross-sectional study
Source: Front Pediatr. 2026 Jun 26;14:1852387. doi: 10.3389/fped.2026.1852387 (PMC13349896; doi:10.3389/fped.2026.1852387)
Supplement: Supplementary file 1 [file Table1.docx]

Supplementary Material

# Supplementary Tables

Table S1 Items of the questionnaire (Total 27).

| Items | |
| --- | --- |
| Basic Information | Name of subject |
|  | Gender |
|  | Age |
| Family Information | Father's education background |
|  | Mother's education background |
|  | Annual family income |
|  | Only child |
| Fertility information | Childbearing age |
|  | Gestational age |
|  | Mode of birth |
|  | Antibiotics at birth |
|  | Mother with FA |
|  | Feeding pattern |
| Environment | Residence has intersection or factory |
|  | Residential floor |
|  | Move into a new house |
|  | Indoor plant |
|  | Pet feeding |
|  | Any smokers at home |
| Habits of life | Particular about food |
|  | Annoying types of food |
| Food Allergy Information | Self-reported FA |
|  | Medically diagnosed FA |
|  | Symptoms of FA |
|  | Time of FA symptoms |
|  | Types of allergens for FA |
| Attachments | Medical certificate |

FA, food allergy

Table S2 The types of food allergens in the questionnaire.

| Types of food | Food |
| --- | --- |
| Seafood | Shrimp; Crab; Fish; Shellfish |
| Fruits | Mango; Banana; Tomato; Pineapple; Peach; Fig |
| Cow's milk | Cow’s milk; Infant formula; Yogurt; Cheese; Butter |
| Egg | Whole egg, Egg white, Egg yolk |
| Peanuts | / |
| Soybeans | / |
| Nuts | Walnut, Almond, Cashew, Hazelnut, Pistachio |
| Others | Wheat; Asparagus; Eggplants; Puffed food; White fungus; Unknown |

Table S3 The ORs and 95% CI of risk factors associated with FA (Backward stepwise (Likelihood ratio method)).

| Variables | *β* value | SE value | Wald *χ^2^* | OR | 95% CI | | *p* |
| --- | --- | --- | --- | --- | --- | --- | --- |
|  |  |  |  |  | Lower | Upper |  |
| Annual family income (RMB) |  |  |  |  |  |  |  |
| < 150000 |  |  |  |  |  |  |  |
| ≥ 150000 | 1.034 | 0.305 | 11.497 | 2.812 | 1.547 | 5.111 | 0.001* |
| Gestational age (weeks) |  |  |  |  |  |  |  |
| < 37 |  |  |  |  |  |  |  |
| 37 ≤ week < 42 | -3.017 | 1.101 | 7.508 | 0.049 | 0.006 | 0.424 | 0.006* |
| ≥ 42 | -1.040 | 0.412 | 6.363 | 0.353 | 0.157 | 0.793 | 0.012* |
| Mother with FA | 1.145 | 0.376 | 9.297 | 3.143 | 1.505 | 6.561 | 0.002* |
| Particular about food | 0.939 | 0.314 | 8.960 | 2.558 | 1.383 | 4.732 | 0.003* |
| Indoor plant | -0.838 | 0.324 | 6.675 | 0.433 | 0.229 | 0.817 | 0.010* |

**p* < 0.05, FA food allergy, OR odds ratio, CI confidence interval
